# Extended Analysis of Axonal Injuries Detected Using Magnetic Resonance Imaging in Critically Ill Traumatic Brain Injury Patients

**DOI:** 10.1089/neu.2021.0159

**Published:** 2022-01-11

**Authors:** Jonathan Tjerkaski, Harriet Nyström, Rahul Raj, Caroline Lindblad, Bo-Michael Bellander, David W. Nelson, Eric P. Thelin

**Affiliations:** ^1^ Department of Clinical Neuroscience, Department of Physiology and Pharmacology, Karolinska Institutet, Stockholm, Sweden.; ^2^ Department of Neuroradiology, Karolinska University Hospital, Stockholm, Sweden.; ^3^ Department of Neurosurgery, Helsinki University Hospital and University of Helsinki, Helsinki, Finland.; ^4^ Department of Neurosurgery, Department of Physiology and Pharmacology, Karolinska Institutet, Stockholm, Sweden.; ^5^ Department of Section for Perioperative Medicine and Intensive Care, Department of Physiology and Pharmacology, Karolinska Institutet, Stockholm, Sweden.; ^6^ Department of Neurology, Karolinska University Hospital, Stockholm, Sweden.

**Keywords:** diffuse axonal injury, machine learning, magnetic resonance imaging, traumatic axonal injury, traumatic brain injury

## Abstract

Studies show conflicting results regarding the prognostic significance of traumatic axonal injuries (TAI) in patients with traumatic brain injury (TBI). Therefore, we documented the presence of TAI in several brain regions, using different magnetic resonance imaging (MRI) sequences, and assessed their association to patient outcomes using machine learning. Further, we created a novel MRI-based TAI grading system with the goal of improving outcome prediction in TBI. We subsequently evaluated the performance of several TAI grading systems. We used a genetic algorithm to identify TAI that distinguish favorable from unfavorable outcomes. We assessed the discriminatory performance (area under the curve [AUC]) and goodness-of-fit (Nagelkerke pseudo-R^2^) of the novel Stockholm MRI grading system and the TAI grading systems of Adams and associates, Firsching and coworkers. and Abu Hamdeh and colleagues, using both univariate and multi-variate logistic regression. The dichotomized Glasgow Outcome Scale was considered the primary outcome. We examined the MRI scans of 351 critically ill patients with TBI. The TAI in several brain regions, such as the midbrain tegmentum, were strongly associated with unfavorable outcomes. The Stockholm MRI grading system exhibited the highest AUC (0.72 vs. 0.68–0.69) and Nagelkerke pseudo-R^2^ (0.21 vs. 0.14–0.15) values of all TAI grading systems. These differences in model performance, however, were not statistically significant (DeLong test, *p* > 0.05). Further, all included TAI grading systems improved outcome prediction relative to established outcome predictors of TBI, such as the Glasgow Coma Scale (likelihood-ratio test, *p* < 0.001). Our findings suggest that the detection of TAI using MRI is a valuable addition to prognostication in TBI.

## Introduction

Because of its rapid acquisition time and sensitivity to intracranial hemorrhage, computed tomography (CT) is the imaging modality of choice in traumatic brain injury (TBI).^1,2^ There are, however, instances in which the severity of a patient's neurological condition does not coincide with CT findings, a phenomenon that is indicative of traumatic axonal injuries (TAI). Th17e TAI, also referred to as diffuse axonal injuries (DAI), occur as a consequence of angular acceleration-deceleration forces that are exerted on the brain at the time of injury, leading to a shearing of axons.^3^ The TAI have been associated with loss of consciousness, disability, and poor outcomes after TBI.^4–7^ Magnetic resonance imaging (MRI), which exhibits greater sensitivity to TAI than CT, is the main imaging modality for diagnosing TAI.^8^

Adams and associates (1989)^9^ stratified TAI into three grades with increasing severity of outcome, based on post-mortem findings (Table 1). Grade 1 encompasses lesions located in the lobar white matter, grade 2 involves lesions to the corpus callosum, and grade 3 was defined as lesions in the dorsolateral rostral brainstem.^9^ Brainstem TAI were further investigated by Firsching and coworkers (2001),^10^ who found that bilateral brainstem injuries were associated with a poorer prognosis than unilateral brainstem lesions. Abu Hamdeh and colleagues (2017)^11^ further evaluated the importance of lesion location, revealing that TAI detected using susceptibility-weighted imaging (SWI) in the midbrain tegmentum were strongly associated with poor outcomes.

**Table 1.** Grading Systems of Traumatic Axonal Injuries

Adams (1989)		Firsching (2001)		Abu Hamdeh (2017)		
MRI findings^†^	Grade	MRI findings	Grade	MRI findings	Grade	
					Age <30	Age >30
Hemispheric lesions	I	Supratentorial lesions only	I	Hemispheric lesions	Ia	Ib
Corpus callosum lesions	II	Unilateral brainstem lesions at any level (± supratentorial injury)	II	Corpus callosum lesions	IIa	IIb
Brainstem lesions	III	Bilateral lesions of the mesencephalon (± supratentorial injury)	III	Brainstem lesions^*^	IIIa	IIIb
		Bilateral lesions of the pons (± supratentorial injury)	IV	Lesions in the substantia nigra or the mesencephalic tegmentum	IVa	IVb

A summary of classification systems of traumatic axonal injuries detected by magnetic resonance imaging (MRI) in patients with traumatic brain injury (TBI).

† Originally based on histopathological findings. ^*^Except for the lesions in the substantia nigra or the mesencephalic tegmentum region.

Only the brainstem component of the grading system of Adams and associates,^9^ however, consistently proves to be statistically significant in predictive models, which indicates that certain forms of TAI may not be prognostically significant.^12–15^ Further, the grading system of Abu Hamdeh and colleagues^11^was not found to be associated with outcomes in a cohort of patients with TBI having undergone decompressive craniectomy.^16^ The inconsistent performance of existing TAI grading systems suggests that a detailed assessment of their prognostic utility is warranted.

In this study, we documented the presence of TAI in several brain regions, using different MRI sequences, and assessed their association to patient outcomes. Further, we created a novel MRI-based TAI grading system, the Stockholm MRI grading system, with the goal of improving outcome prediction in TBI. We subsequently evaluated the performance of several TAI grading systems with regard to outcome prediction.

## Methods

### Study design

This is a retrospective observational study from the intensive care unit (ICU) at the Karolinska University Hospital (Stockholm, Sweden). The study protocol was approved by the Swedish Ethical Review Authority (#2019-04476).

### Inclusion and exclusion criteria

We included patients with blunt TBI (aged ≥15 years) who were admitted to the ICU after TBI during 2005–2019, who underwent an MRI-examination within the first 28 days of the trauma, and had an outcome assessment at ≥6 months post-trauma (using the Glasgow Outcome Scale {GOS] assessed at a median of 359 days).^17^

### Outcome

The GOS, a measure of long-term functional outcome, was assessed at follow-up appointments or by questionnaires. We defined unfavorable outcome as GOS 1–3 and favorable outcome as GOS 4–5 (dichotomized GOS). Additional outcome measures, such as the extended GOS or neuropsychiatric outcomes, could not be investigated in the present study, because they were not recorded prospectively in our data registries.

### Image analysis

Patients with TBI whose clinical examinations suggest pathology that was not evident on CT scans are referred for MRI at our institution. Study participants were scanned using either a Siemens Avanto (Siemens Healthineers AG, Erlangen, Germany) or a GE Signa (General Electric Company, Boston, MA) scanner at 1.5T. The scanning protocols were similar between the two scanner types but evolved over time to maximize diagnostic accuracy (Supplementary Tables S1–S3). Patients who were admitted during the period 2018–2019 (*n* = 24) were scanned using a GE Signa scanner at 3T.

We defined TAI as either (1) hypointensities on the T2*-weighted gradient echo (T2*GRE) and SWI sequences, (2) an increase in signal intensity using T2-weighted fluid attenuated inversion recovery (FLAIR), or (3) restricted diffusion on diffusion-weighted imaging (DWI) (Supplementary Fig. S1–S3).^18^ The T2*GRE was initially the MRI sequence that was used to detect hemorrhage, but it was replaced by SWI in 2010. We therefore combined the results obtained using SWI and T2*GRE, collectively referring to them as the susceptibility-sensitive sequences.

We documented the presence of TAI detected using MRI in several anatomical locations (Supplementary Table S4). We categorized TAI as either present or absent in each of the assessed brain regions. We documented the percentage of unfavorable outcome associated with TAI in each of the investigated anatomical locations, as a proxy for the severity of these lesions. The same protocol was repeated using each of the MRI sequences that were included in this study.

For comparative purposes, we assessed the MRI-based TAI grading systems of Adams and associates,^9^ Firsching and coworkers,^10^ and Abu Hamdeh and colleagues.^11,19–22^ We did not examine the grading system of Mannion and associates,^23^ because the time from ICU admission until the MRI examination was expected to differ substantially between this study and that of Mannion and associates (median 1 day).^23^ Because the grading systems of Adams and associates^9^ and Firsching and coworkers^10^ do not state explicitly which MRI pulse sequences are to be used, we used FLAIR, DWI, and the susceptibility-sensitive sequences and followed the same definition of TAI that we used for the current study.

In case of disagreement between the different MRI pulse sequences concerning grading, we selected the most severe grade. We used the same protocol when assessing grades I–III of the grading system of Abu Hamdeh and colleagues.^11^ When assessing grade IV using the grading system of Abu Hamdeh and colleagues,^11^ we used T2* GRE if SWI was unavailable. Patients who did not meet the criteria for any of the categories described in the grading systems of Adams and associates,^9^ Firsching and coworkers,^10^ or Abu Hamdeh and colleagues,^11^ respectively, were marked as “grade 0” for that particular grading system.

In addition, we computed the Rotterdam CT score using admission CT scans.^20^ We performed all assessments of CT and MRI scans blinded to the patients' mechanism of injury, clinical status, and outcome. Author HN, a neuroradiologist with more than 20 years of experience, was responsible for image analysis.

### Statistical analysis

To account for possible selection bias, we compared patient characteristics between patients with TBI who have undergone an MRI examination with those who have not done so at our institution. We assessed group differences in continuous and categorical variables using the Mann-Whitney *U* test and the chi-square test, respectively. We assessed group differences in ordinal data using the Cochran-Armitage trend test.

We imputed missing data using multi-variate imputation by chained equations (MICE).^24^ We split the data into a training dataset (2/3 of the data) and a test dataset (1/3 of the data). These datasets contained information regarding the presence of TAI in several brain regions detected using FLAIR, DWI, or the susceptibility-sensitive sequences, respectively. We used a genetic algorithm (GA) on the training dataset, to examine various configurations of TAI, to identify that which has the best performance in predicting functional outcome. A GA is a search algorithm and an optimization method that is based on the concept of natural selection.^25^ In each iteration of the GA, we assessed model performance using a desirability function,^26^ which aimed to maximize discrimination while simultaneously penalizing excessively complex models, thereby limiting the risk of overfitting.

Next, we used a random forest model to internally validate the utility of the variables that were selected by the GA in the independent test dataset. The random forest model, which was initially trained on the training dataset, included the TAI variables identified by the GA, as well as several known outcome predictors, to assess whether the MRI characteristics selected by the GA are capable of providing prognostic information in excess of established outcome predictors in TBI. The included outcome predictors were age, admission Glasgow Coma Scale (GCS) score, and pupillary light responsiveness (“core variables”).^27^ The dichotomized GOS was used as the dependent variable.

We used the area under the receiver operating characteristic curve (AUC) to assess the ability of the random forest model to discriminate between favorable and unfavorable outcomes in the test dataset. The predictive power of the TAI variables that were selected by the GA was assessed by computing the mean decrease in Gini impurity, a measure of variable importance, using the random forest model. For comparative purposes, we created an additional random forest model, including only the core variables.

Further details regarding feature selection and machine learning are provided in the supplementary materials (see Supplementary Information).

We used a GA to obtain a parsimonious solution to outcome prediction in TBI, based on various configurations of TAI detected using DWI, FLAIR, or the susceptibility-weighted sequences in different brain regions. The result of the GA consisted of a single combination of TAI lesion types, which was found to have the best performance in discriminating between favorable and unfavorable outcomes. The brain regions that were represented among the TAI variables that were selected by the GA constituted the basis for the novel Stockholm MRI grading system. In addition, we used the mean decrease in Gini impurity and the percentage of unfavorable outcomes attributed to each of the selected TAI variables to stratify TAI based on their severity.

Fine-tuning of the Stockholm MRI grading system was ultimately performed using multi-variate logistic regression, to optimize the configuration of TAI in each stratum of the Stockholm MRI grading system. We examined model performance by assessing the discrimination between favorable and unfavorable outcomes (AUC). The version of the Stockholm MRI regarding system that resulted in the best performing multi-variate logistic regression model constituted the final version of the Stockholm MRI grading system.

We assessed the performances of the Stockholm MRI grading system and the previously described grading systems of Adams and associates,^9^ Firsching and coworkers,^10^ and Abu Hamdeh and colleagues^11^ using both univariate and multi-variate logistic regression. We examined the performance of all grading systems in isolation and following the addition of the core variables and the Rotterdam CT score. Other parameters, such as sex or laboratory values, were not included in these regression models, because their prognostic significance is less established.^20,27,28^

We evaluated all logistic regression models using the Nagelkerke pseudo-R^2^, AUC, and the Akaike information criterion (AIC), with dichotomized GOS as the dependent variable. The grading system of Abu Hamdeh and colleaues^11^ was used without age group separation, because age was included as a covariate in all multi-variate logistic regression models.

We used the likelihood ratio test to examine whether the addition of MRI-based TAI grading systems improved the performance of multi-variate logistic regression models. We used the DeLong test to compare different TAI grading systems with regard to the discrimination between favorable and unfavorable outcomes. We considered *p* values <0.05 to be statistically significant.

We used R (version 3.6.0, R Foundation for Statistical Computing, Vienna, Austria) to perform the statistical analysis.^29^

## Results

### Demographics

Of 1578 admitted patients with TBI, 351 (22%) fulfilled the inclusion criteria (Supplementary Fig. S4). Patients who underwent MRI were younger, had lower GCS scores, longer ICU lengths of stay, and had worse outcomes than patients with TBI who did not undergo MRI at our institution (Table 2). Motor vehicle accidents were more common in patients with unfavorable outcomes (Supplementary Table S5). Missing entries amounted to fewer than 4% of all acquired data (Supplementary Table S6).

**Table 2.** Demographics

	MRI scan performed (*n* = 351)	MRI scan not performed (*n* = 1201)	*p*
Age (years)			< 0.001^1^
Mean (SD)	43.8 (18.6)	50.2 (18.4)	
Range (Min – Max)	15.0–82.0	15.0–92.0	
Gender			0.991^3^
Male	260 (74.1%)	890 (74.1%)	
Female	91 (25.9%)	311 (25.9%)	
GCS			< 0.001^2^
Median (Q1–Q3)	4.0 (3.0–7.0)	12.0 (6.0–14.0)	
Range (Min – Max)	3.0–15.0	3.0–15.0	
Pupillary response			< 0.001^4^
Unilaterally unresponsive	34 (9.9%)	37 (3.2%)	
Bilaterally unresponsive	66 (19.2%)	96 (8.3%)	
Responsive	243 (70.8%)	1029 (88.6%)	
Glasgow Outcome Scale			< 0.001^4^
1	38 (10.8%)	158 (13.2%)	
2	9 (2.6%)	2 (0.2%)	
3	136 (38.7%)	239 (19.9%)	
4	106 (30.2%)	397 (33.1%)	
5	62 (17.7%)	405 (33.7%)	
NCCU stay duration (days)			< 0.001^2^
Median (Q1–Q3)	14.0 (8.0–20.6)	1.3 (0.0–4.6)	
Range (Min – Max)	0.0–53.0	0.0–44.3	
Time until the MRI examination (days)			
Median (Q1–Q3)	7.0 (4.0–13.0)		
Range (Min – Max)	0.0–28.0		

MRI, magnetic resonance imaging; SD, standard deviation; GCS, Glasgow Coma Scale; NCCU, neurological critical care unit .

1: Student *t* test. 2: Mann-Whitney *U* test. 3: Pearson chi-square test 4: Cochran-Armitage trend test for ordinal variables.

### Severity of TAI

In 73% of the study participants, TAI was detected. In general, lesions with restricted diffusion on DWI were more severe but less frequent than those detected using FLAIR or the susceptibility-sensitive sequences (Table 3). THE TAI that were associated with unfavorable outcomes include bilateral TAI in the thalamus, midbrain and pons (on average 87%, 90%, and 97% unfavorable outcomes, respectively), TAI in the posterior limb of the internal capsule (on average 83–91% unfavorable outcomes), and TAI in the midbrain tegmentum (on average 81% unfavorable outcomes).

**Table 3.** Severity of Traumatic Axonal Injuries

Diffusion-weighted imaging			Fluid attenuated inversion recovery			Susceptibility-sensitive sequences		
Lesion type	*n*	Unfavorable outcome (%)	Lesion type	*n*	Unfavorable outcome (%)	Lesion type	*n*	Unfavorable outcome (%)
Basal ganglia								
Unilateral	5	100	Unilateral	18	78	Unilateral	44	61
Bilateral	0	-	Bilateral	3	100	Bilateral	7	71
Corpus Callosum								
Trunk	23	83	Trunk	57	67	Trunk	68	71
Splenium	82	63	Splenium	111	68	Splenium	88	69
Genu and Rostrum	23	78	Genu and Rostrum	27	78	Genu and Rostrum	41	59
Internal capsule								
Unilateral	18	89	Unilateral	36	89	Unilateral	40	80
Bilateral	3	100	Bilateral	11	100	Bilateral	15	87
Midbrain								
Unilateral	35	86	Unilateral	55	69	Unilateral	43	70
Tegmentum	24	100	Tegmentum	57	81	Tegmentum	58	83
Tectum	9	89	Tectum	21	86	Tectum	15	67
Cerebral peduncles	18	89	Cerebral peduncles	32	84	Cerebral peduncles	30	73
Bilateral	11	100	Bilateral	24	96	Bilateral	39	87
Pons								
Ventral	8	100	Ventral	25	84	Ventral	26	96
Unilateral	17	88	Unilateral	33	67	Unilateral	25	68
Dorsal	13	85	Dorsal	33	76	Dorsal	35	77
Bilateral	4	100	Bilateral	15	100	Bilateral	22	95
Subcortical								
Unilateral	16	62	Unilateral	51	49	Unilateral	67	46
Bilateral	8	62	Bilateral	57	72	Bilateral	106	60
Thalamus								
Unilateral	18	83	Unilateral	39	72	Unilateral	37	68
Bilateral	4	100	Bilateral	16	88	Bilateral	29	90
No detected traumatic axonal injuries								
Total	206	40	Total	128	38	Total	106	41

Interestingly, unilateral TAI detected using FLAIR and the susceptibility-sensitive sequences in the midbrain and the pons were similar in terms of severity as, for instance, TAI in the splenium of the corpus callosum (67–70% and 68–69% unfavorable outcomes, respectively). Patients in whom TAI remained undetected exhibited unfavorable outcomes in approximately 40% of cases, likely because of the contribution of other lesions (e.g., cerebral contusions, extra-axial hematomas, or cerebral edema).

The proportion of patients in whom TAI was detected diminished over time, with non-hemorrhagic injuries being affected to a greater extent than hemorrhagic TAI (Supplementary Fig. S5). These results coincide with those of previous studies, suggesting that non-hemorrhagic TAI become less conspicuous on MRI as time passes.^30^

### Feature selection and machine learning

**FIG. 1.**
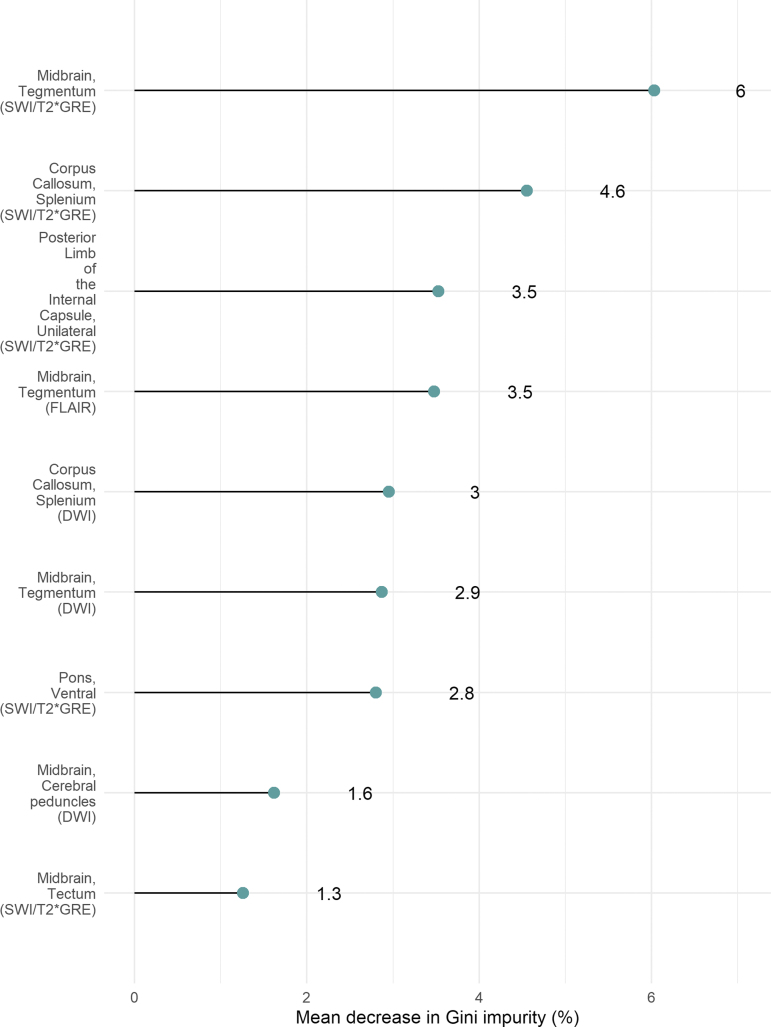
Variable importance. The predictive power of the traumatic axonal injuries (TAI) variables that were selected by the genetic algorithm (GA) was assessed by computing the mean decrease in Gini impurity, a measure of variable importance, using the random forest model that consisted of the core variables and TAI. Color image is available online.

**FIG. 2.**
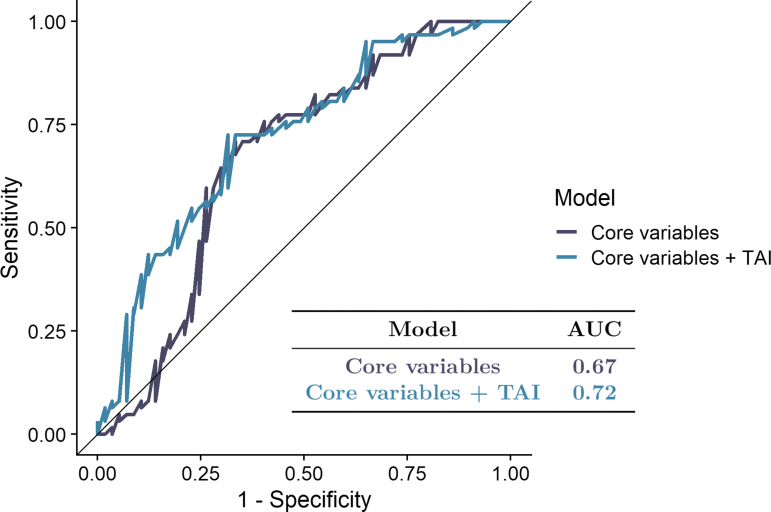
Random forest. Results for the random forest model that was fitted using the traumatic axonal injuries (TAI) selected by the genetic algorithm and the core variables, as well as another random forest model that was fitted using the core variables only. Color image is available online.

We found that TAI detected using the susceptibility-sensitive sequences in the midbrain tegmentum, the splenium of the corpus callosum, and the posterior limb of the internal capsule were the most important outcome predictors among the TAI variables that were selected by the GA (Fig. 1). The addition of the features selected by the GA improved the predictive performance of the random forest model, resulting in an increase of the AUC from 0.67 to 0.72 (Fig. 2).

### Stockholm MRI grading system

The final version of the Stockholm MRI grading system consists of grades I–IV, in escalating clinical severity (Table 4). Although not among the variables chosen by the GA, we found that the addition of thalamic TAI improved the predictive performance of multi-variate logistic regression models. Therefore, we chose to incorporate thalamic TAI into the Stockholm MRI grading system.

**Table 4.** Stockholm Magnetic Resonance Imaging Grading System

MRI findings^**a**^	Grade	Unfavorable outcomes
• Bilateral TAI in the pons	IV	97%
• TAI in the midbrain tegmentum (unilateral or bilateral) and/or • Bilateral TAI in the thalamus and/or • TAI in the posterior limb of the internal capsule (unilateral or bilateral)	III	74%
• TAI in the corpus callosum and/or • Unilateral TAI in the thalamus and/or • Unilateral TAI in the pons and/or • Midbrain TAI located outside of the tegmentum region (unilateral or bilateral)	II	40%
• All brain trauma patients who do not meet the requirements of grades II-IV.^**b**^	I	28%

^a^ The different grades are mutually exclusive, where the highest possible grade using any of the described magnetic resonance imaging (MRI) pulse sequences is to be given precedence. A single one of the lesion types included within e.g., grade II or grade III is sufficient for a patient to be classified as such, and the presence of more than one of those lesion types does not influence grading according to the Stockholm MRI grading system. The grading system is only applicable to MRI examinations of adult patients with traumatic brain injury from blunt trauma performed within a period of 28 days post-trauma. ^b^Grade I of the Stockholm MRI grading system includes both patients with traumatic axonal injuries (TAI) in regions outside those specified in the instructions of grades II-–V, as well as patients in whom TAI was not detected. *Abbreviations*:

### Comparison of MRI-based TAI grading systems

Four different MRI-based TAI-grading systems were compared using both univariate and multi-variate logistic regression (Table 5). The addition of each TAI grading system to the core variables and the Rotterdam CT score improved model performance (likelihood ratio test, *p* < 0.01), indicating that MRI-based TAI grading systems improve the accuracy of outcome prediction in patients with TBI. Logistic regression models that contained the Stockholm MRI grading system exhibited the highest AIC, AUC and Nagelkerke pseudo-R^2^ values of all the studied TAI grading systems. We observed, however, no statistically significant differences in discriminatory performance when comparing the TAI grading systems with each other (Supplementary Table S7). The corresponding ROC curves and calibration plots are provided in the supplementary materials (Supplementary Fig. S6 and S7).

**Table 5.** Outcome Prediction Based on Traumatic Axonal Injuries Detected Using Magnetic Resonance Imaging in Patients with Traumatic Brain Injury

Model	Pseudo-R^2^	AIC	AUC
Core	0.24	426	0.75
Rotterdam CT-score	0.08	476	0.63
MRI grading system of Adams^9^	0.15	453	0.68
MRI grading system of Firsching^10^	0.14	457	0.68
MRI grading system of Abu Hamdeh^11^	0.15	455	0.69
Stockholm MRI grading system	0.21	433	0.72
Core + Rotterdam CT-score	0.28	424	0.77
Core + MRI grading system of Adams^9^	0.32	405	0.79
Core + MRI grading system of Firsching^10^	0.32	406	0.79
Core + MRI grading system of Abu Hamdeh^11^	0.33	405	0.79
Core + Stockholm MRI grading system	0.38	384	0.82
Core + Rotterdam CT-score + MRI grading system of Adams^9^	0.35	404	0.80
Core + Rotterdam CT-score + MRI grading system of Firsching^10^	0.36	403	0.81
Core + Rotterdam CT-score + MRI grading system of Abu Hamdeh^11^	0.36	403	0.81
Core + Rotterdam CT-score + Stockholm MRI grading system	0.41	383	0.83

Pseudo-R^2^, Nagelkerke pseudo-R^2^; AIC, Akaike information criterion; AUC, area under the receiver operating characteristic curve; Core, age, pupillary reactivity, and Glasgow Coma Scale; CT, computed tomography; MRI, magnetic resonance imaging.

## Discussion

We performed a thorough investigation of TAI detected using MRI in a large retrospective cohort. We utilized the evolutionary algorithm GA to identify TAI associated with long-term functional outcome after TBI. We observed no statistically significant differences between the investigated TAI grading systems with regard to the discrimination between favorable and unfavorable outcomes. In contrast to other studies,^12,16^ we found that all investigated TAI grading systems resulted in statistically significant improvements in the performance of logistic regression models. In summary, our results suggest that the detection of TAI using MRI improves prognostication in critically ill patients with TBI.

Although different, the investigated TAI grading systems also share many similarities, because they all examine the same pathophysiological entity. Our results suggest that the differences between the investigated TAI grading systems are not substantial enough for there to be a statistically significant difference in their prognostic capability.

The study cohort had lower GCS scores and longer ICU lengths of stay than the ICU-treated TBI population as a whole. These findings suggest that there may have been bias in the inclusion of study participants, which is likely because of the selective nature of MRI referrals at our institution.

The results of this study suggest that there is a difference in terms of severity among different types of brainstem TAI, which has been proposed previously by multiple sources.^31,32^ This is in stark contrast to the grading system of Adams and associaes,^9^ which regards all brainstem TAI in an identical manner.^9^

Our findings suggest that TAI detected using the susceptibility-sensitive sequences in the midbrain tegmentum was the most important outcome predictor of all TAI, which corroborates the results of Abu Hamdeh and colleagues (2017).^11^ In addition, our results indicate that all lesions in the midbrain tegmentum are associated with unfavorable outcomes, regardless of the MRI pulse sequence used to detect them.

We found that bilateral TAI in the pons was the most severe of all lesion types, likely because of the involvement of the pontine reticular formation.^32^ These results corroborate the observations of Firsching and coworkers.^10^ Although we observed that bilateral brainstem TAI were more severe than unilateral brainstem TAI, it is entirely possible that these findings are confounded by differences in lesion volume, because bilateral injuries tend to be larger than unilateral injuries.^32^ One might speculate that volumetric measurements using automated lesion segmentation tools might further improve outcome prediction based on MRI in TBI.

The Stockholm MRI grading system is the first TAI grading system to incorporate TAI in the thalamus and the posterior limb of the internal capsule. Similar to brainstem injuries, lesions in the thalamus have also been associated with impaired consciousness, which might explain the association between thalamic lesions and unfavorable functional outcomes in the setting of TBI.^33^ Injuries to the posterior limb of the internal capsule, an integral part of the corticospinal tract, contribute to motor weakness after TBI,^34^ a state that likely impairs an individual's ability to reach a favorable functional outcome.

Limitations, which largely stem from the retrospective nature of this study, include the fact that different scanning protocols and MRI scanners were used at different time points throughout the duration of this study, as well as fact that there was considerable variability in the time at which the study participants were referred for MRI. Nevertheless, it can be expected that the available MRI scanners will vary across different medical institutions and that the scanning protocols used will evolve over time. Likewise, inevitably there will be a certain degree of variability in the time at which critically ill patients with TBI can be considered sufficiently stable for transportation to the radiology department. Thus, we were able to evaluate the prognostic significance of TAI in an authentic real-world setting.

## Conclusions

Our findings suggest that the detection of TAI using MRI is a valuable addition to prognostication in patients with TBI.

## Supplementary Material

Supplemental data

Supplemental data

Supplemental data

Supplemental data

Supplemental data

Supplemental data

Supplemental data

Supplemental data

Supplemental data

Supplemental data

Supplemental data

Supplemental data

Supplemental data

Supplemental data

Supplemental data
