# Supplementary material for: Extended Analysis of Axonal Injuries Detected Using Magnetic Resonance Imaging in Critically Ill Traumatic Brain Injury Patients
Source: J Neurotrauma. 2022 Jan 11;39(1-2):58–66. doi: 10.1089/neu.2021.0159 (PMC8785713; doi:10.1089/neu.2021.0159)
Supplement: Supplemental data [file Supp_TableS1.docx]

| scanner | Time period | TR (ms) | TE (ms) | TI (ms) | Flip angle |
| --- | --- | --- | --- | --- | --- |
| GE Signa (1.5T) | 2005-2006 | 11002 | 145 | 2200 | 90° |
|  | 2006-2008 | 9002 | 147 | 2200 | 90° |
|  | 2008-2010 | 9002 | 158 | 2200 | 90° |
| Siemens Avanto (1.5T) | 2010-2018 | 9000 | 119 | 2500 | 150° |
| GE Signa (3T) | 2018-2019 | 8000 | 126 | 2092 | 90° |

**Supplemental Table 1.** **MRI protocol for the FLAIR sequence.**

A table summarising the parameters used for the Fluid attenuating inversion recovery sequence. Abbreviations: FLAIR = Fluid attenuating inversion recovery, MRI = Magnetic Resonance imaging, GE = General Electric, TR = Repetition time, TE = Echo time.
