# Supplementary material for: Extended Analysis of Axonal Injuries Detected Using Magnetic Resonance Imaging in Critically Ill Traumatic Brain Injury Patients
Source: J Neurotrauma. 2022 Jan 11;39(1-2):58–66. doi: 10.1089/neu.2021.0159 (PMC8785713; doi:10.1089/neu.2021.0159)
Supplement: Supplemental data [file Supp_TableS3.docx]

| scanner | sequence | Time period | TR (ms) | TE (ms) | Flip angle |
| --- | --- | --- | --- | --- | --- |
| GE Signa (1.5T) | T2*GRE | 2005-2006 | 440 | 15 | 15° |
|  |  | 2005-2008 | 460 | 15 | 15° |
|  |  | 2007-2010 | 700 | 30 | 20° |
| Siemens Avanto (1.5T) | SWI | 2010-2018 | 49 | 40 | 15° |
| GE Signa (3T) |  | 2018-2019 | 30 | 20 | 15° |
|  |  | 2018-2019 | 76 | 48 | 25° |

**Supplemental Table 3.** **MRI protocols for the susceptibility-sensitive sequences.**

A table summarising the parameters used for the susceptibility sensitive sequences. Abbreviations: T2*GRE = T2*-weighted gradient echo, SWI = Susceptibility weighted imaging, MRI = Magnetic Resonance imaging, GE = General Electric, TR = Repetition time, TE = Echo time.
