# Supplementary material for: Extended Analysis of Axonal Injuries Detected Using Magnetic Resonance Imaging in Critically Ill Traumatic Brain Injury Patients
Source: J Neurotrauma. 2022 Jan 11;39(1-2):58–66. doi: 10.1089/neu.2021.0159 (PMC8785713; doi:10.1089/neu.2021.0159)
Supplement: Supplemental data [file Supp_FigS1.docx]

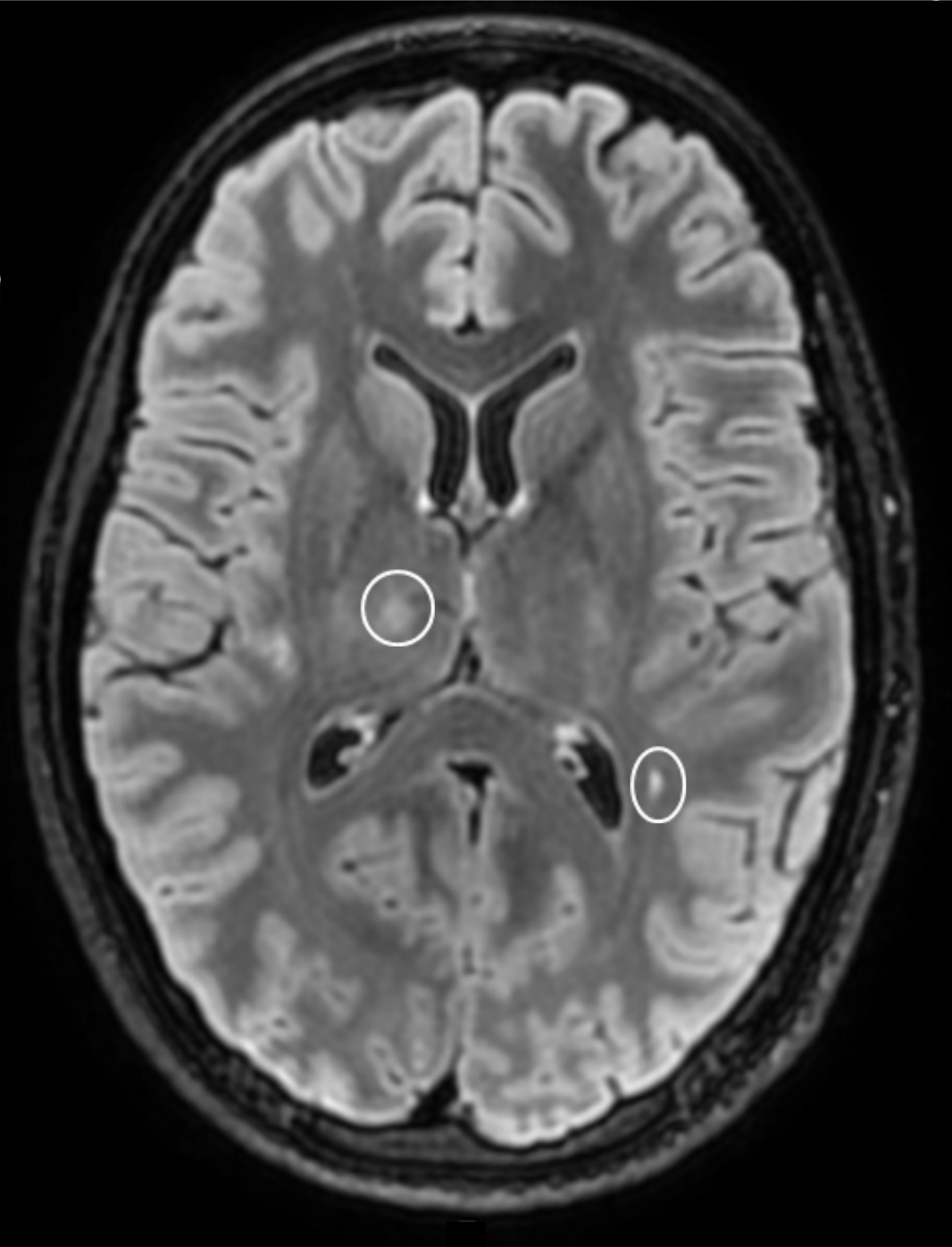


Supplemental Figure 1. Fluid attenuated inversion recovery.

Traumatic axonal injuries detected using the Fluid attenuated inversion recovery (FLAIR) magnetic resonance imaging (MRI) pulse sequence in the right thalamus and the left optic radiation, marked with white circles.
