# Supplementary material for: Extended Analysis of Axonal Injuries Detected Using Magnetic Resonance Imaging in Critically Ill Traumatic Brain Injury Patients
Source: J Neurotrauma. 2022 Jan 11;39(1-2):58–66. doi: 10.1089/neu.2021.0159 (PMC8785713; doi:10.1089/neu.2021.0159)
Supplement: Supplemental data [file Supp_FigS3.docx]

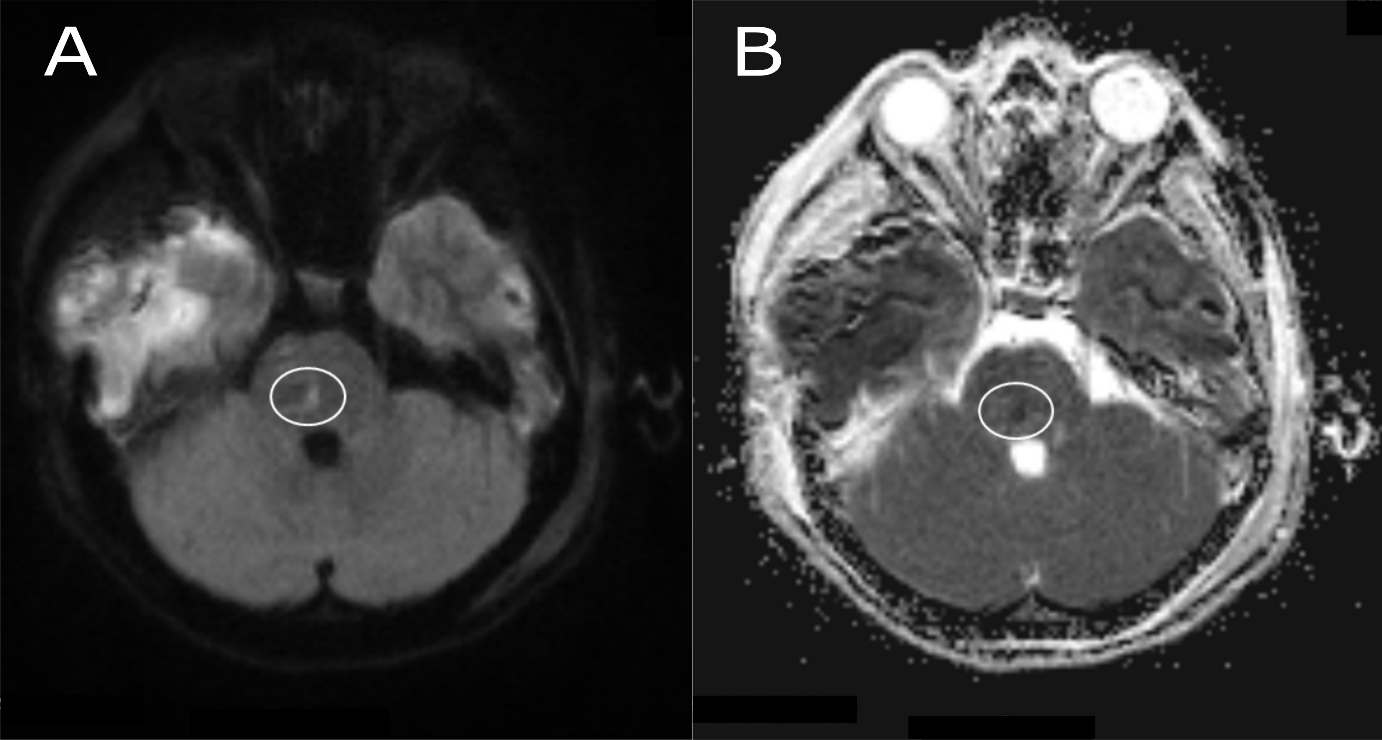


Supplemental Figure 3. Diffusion-weighted imaging.

Traumatic axonal injuries exhibiting a pattern of restricted diffusion in the dorsal Pons, identified by a characteristic hyperintensity on the isotropic diffusion map (A) and a concurrent hypointensity on the Apparent diffusion coefficient (ADC)-map (B).
