# Supplementary material for: Extended Analysis of Axonal Injuries Detected Using Magnetic Resonance Imaging in Critically Ill Traumatic Brain Injury Patients
Source: J Neurotrauma. 2022 Jan 11;39(1-2):58–66. doi: 10.1089/neu.2021.0159 (PMC8785713; doi:10.1089/neu.2021.0159)
Supplement: Supplemental data [file Supp_TableS4.docx]

| **Anatomical location** | **Type of injury** |
| --- | --- |
| Lobar white matter | Unilateral, Bilateral. |
| Corpus callosum | Splenium, Body and Genu. |
| Basal ganglia | Unilateral, Bilateral. |
| Posterior limb of the internal capsule | Unilateral, Bilateral. |
| Thalamus | Unilateral, Bilateral. |
| Midbrain | Unilateral, Bilateral, Cerebral peduncles,  Tegmentum, Tectum. |
| Pons | Unilateral, Bilateral, Ventral, Dorsal. |

Supplemental Table 4. Radiological assessment protocol.

A list of anatomical locations and their sub-categories in which traumatic axonal injuries detected using magnetic resonance imaging were documented.
