# Supplementary material for: Extended Analysis of Axonal Injuries Detected Using Magnetic Resonance Imaging in Critically Ill Traumatic Brain Injury Patients
Source: J Neurotrauma. 2022 Jan 11;39(1-2):58–66. doi: 10.1089/neu.2021.0159 (PMC8785713; doi:10.1089/neu.2021.0159)
Supplement: Supplemental data [file Supp_FigS4.docx]

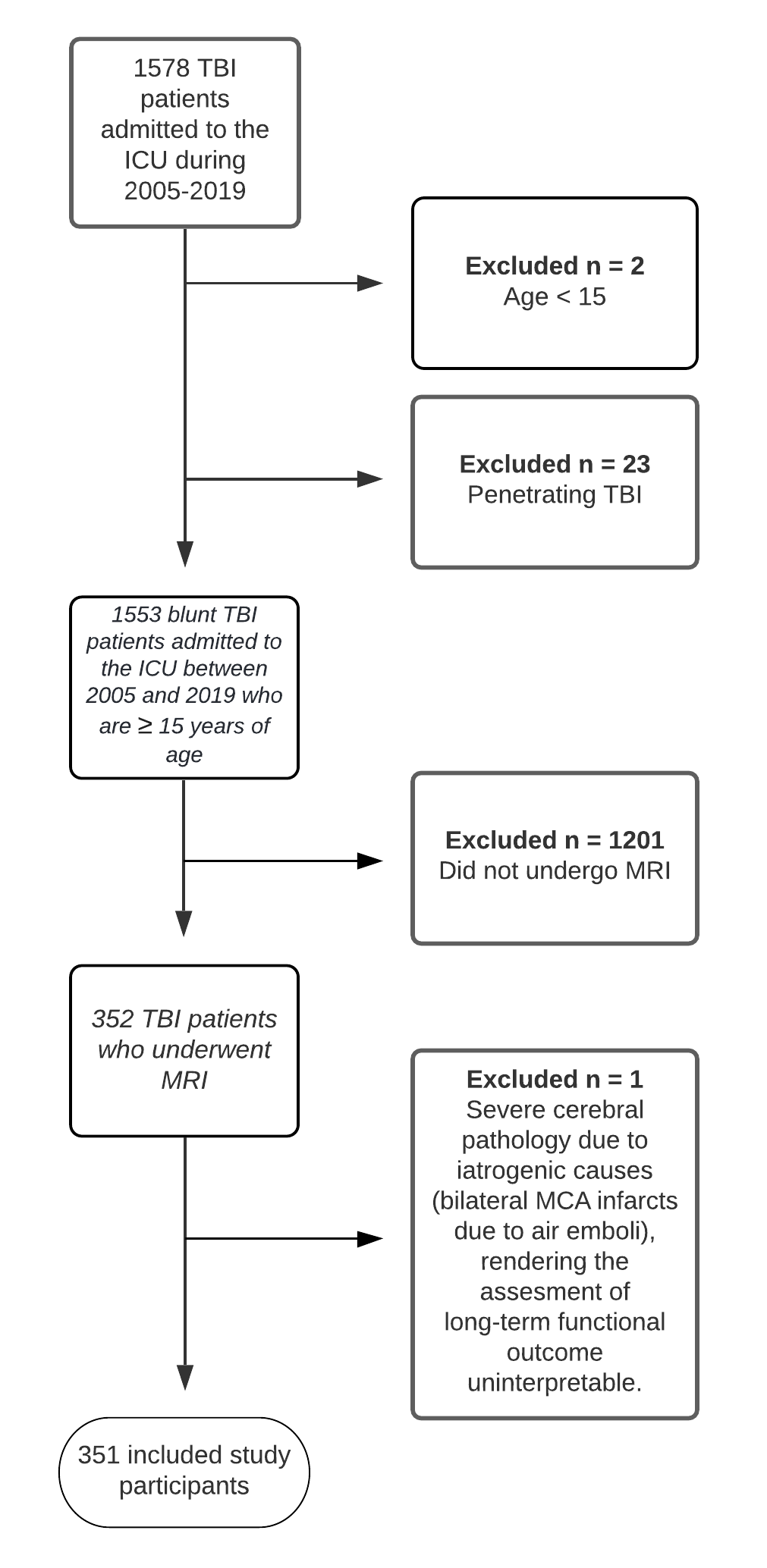


**Supplemental Figure 4. Flowchart of patient inclusion and exclusion**

Diagram describing the inclusion and exclusion of study participants, according to the inclusion and exclusion criteria mentioned in the methodology section of this article.
