# Supplementary material for: Extended Analysis of Axonal Injuries Detected Using Magnetic Resonance Imaging in Critically Ill Traumatic Brain Injury Patients
Source: J Neurotrauma. 2022 Jan 11;39(1-2):58–66. doi: 10.1089/neu.2021.0159 (PMC8785713; doi:10.1089/neu.2021.0159)
Supplement: Supplemental data [file Supp_TableS5.docx]

|  | **Unfavourable (N=183)** | **Favourable (N=168)** |
| --- | --- | --- |
| **Cause of injury**^1^ | n (%) | n (%) |
| Assault with blunt object | 14 (7.7%) | 22 (13.1%) |
| High_energy_fall | 41 (22.4%) | 46 (27.4%) |
| Low energy fall | 29 (15.8%) | 23 (13.7%) |
| Other | 16 (8.7%) | 7 (4.2%) |
| Traffic accident: Bicycle | 8 (4.4%) | 17 (10.1%) |
| Traffic accident: Motorcycle | 13 (7.1%) | 17 (10.1%) |
| Traffic accident: MVA | 34 (18.6%) | 15 (8.9%) |
| Traffic accident: Other | 11 (6.0%) | 10 (6.0%) |
| Traffic accident: Pedestrian | 17 (9.3%) | 11 (6.5%) |

**Supplemental table 5. Cause of injury:** Causes of injury for patients with unfavourable and favourable outcomes in the MRI cohort, respectively. Abbreviations: MRI = Magnetic resonance imaging, MVA = Motor Vehicle Accident. Etiology was defined in accordance with the Utstein template (see reference below).

1. Ringdal, K.G., Coats, T.J., Lefering, R., Bartolomeo, S. Di, Steen, P.A., Røise, O., Handolin, L., and Lossius, H.M. (2008). The Utstein template for uniform reporting of data following major trauma: A joint revision by SCANTEM, TARN, DGU-TR and RITG. Scand. J. Trauma, Resusc. Emerg. Med. 2008 161 16, 1–19.
