# Supplementary material for: Extended Analysis of Axonal Injuries Detected Using Magnetic Resonance Imaging in Critically Ill Traumatic Brain Injury Patients
Source: J Neurotrauma. 2022 Jan 11;39(1-2):58–66. doi: 10.1089/neu.2021.0159 (PMC8785713; doi:10.1089/neu.2021.0159)
Supplement: Supplemental data [file Supp_TableS6.docx]

| **Variable** | **Missing** | **Proportion** |
| --- | --- | --- |
| Computed tomography | 29 | 8 % |
| MRI: FLAIR sequence | 17 | 5 % |
| MRI: Susceptibility-sensitive sequences | 14 | 4 % |
| MRI: DWI sequence | 9 | 3 % |
| Pupillary reactivity | 8 | 2 % |
| **Supplemental Table 6. Quantity and proportion of missing values.** A summary of all the missing data. Note, only variables which included missing data are shown. The remainder of the data which was used in this study contained no missing entries. Abbreviations: DWI = Diffusion Weighted Imaging, FLAIR = Fluid Attenuated Inversion Recovery, MRI = Magnetic Resonance imaging. | | |
