# Supplementary material for: Extended Analysis of Axonal Injuries Detected Using Magnetic Resonance Imaging in Critically Ill Traumatic Brain Injury Patients
Source: J Neurotrauma. 2022 Jan 11;39(1-2):58–66. doi: 10.1089/neu.2021.0159 (PMC8785713; doi:10.1089/neu.2021.0159)
Supplement: Supplemental data [file Supp_FigS5.docx]

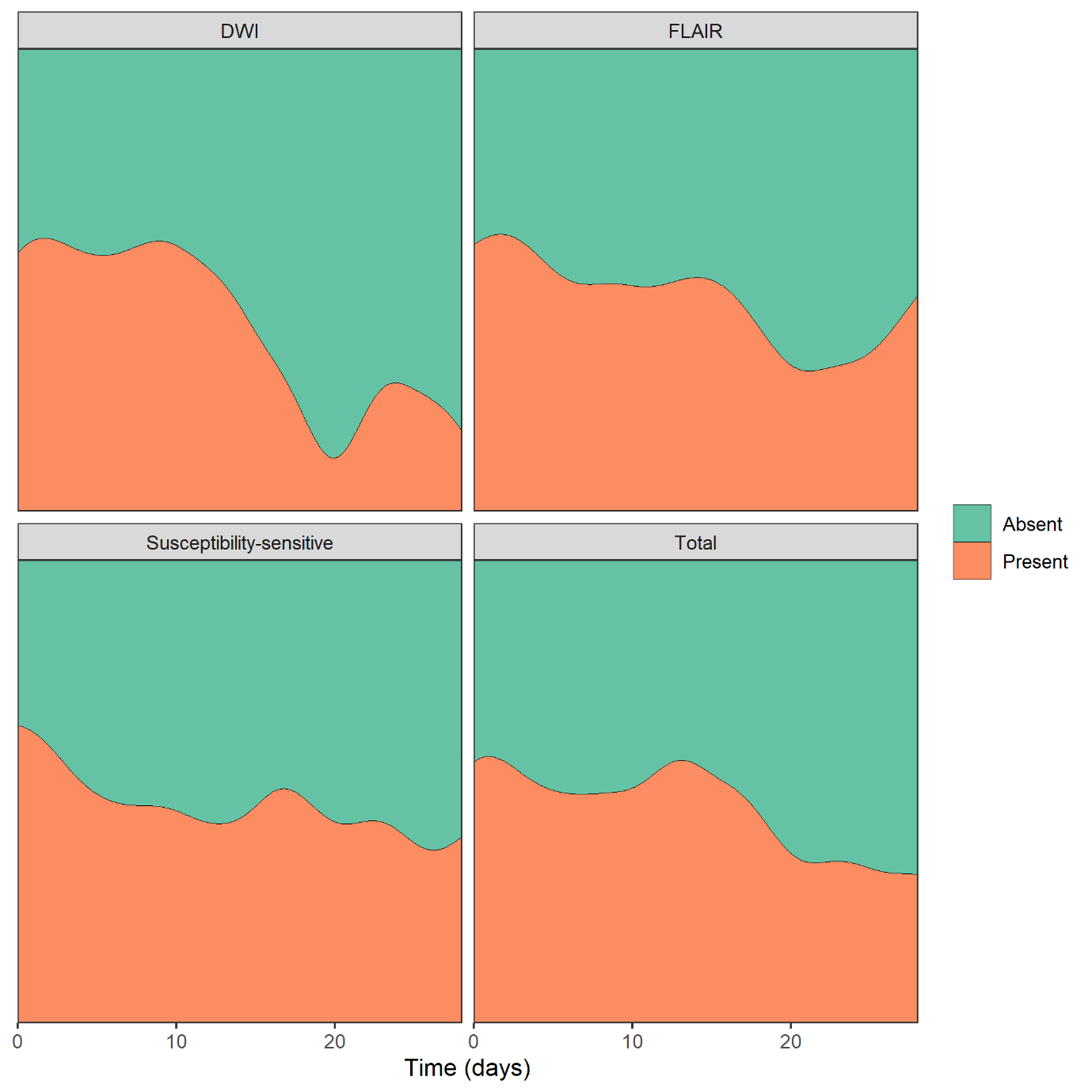


Supplemental Figure 5. Conditional density plot of TAI detected over time.

The proportion of patients in whom TAI is detected diminishes over time for all MRI sequences, with non-haemorrhagic TAI being affected to a greater extent than haemorrhagic TAI. DWI = Diffusion-weighted imaging, FLAIR = Fluid-attenuated inversion recovery.
