# Supplementary material for: Extended Analysis of Axonal Injuries Detected Using Magnetic Resonance Imaging in Critically Ill Traumatic Brain Injury Patients
Source: J Neurotrauma. 2022 Jan 11;39(1-2):58–66. doi: 10.1089/neu.2021.0159 (PMC8785713; doi:10.1089/neu.2021.0159)
Supplement: Supplemental data [file Supp_TableS7.docx]

| **Comparison** | **p value** |
| --- | --- |
| MRI grading system of Adams et al. vs. MRI grading system of Firsching et al. | 0.551 |
| MRI grading system of Adams et al. vs. MRI grading system of Abu Hamdeh et al. | 0.890 |
| MRI grading system of Adams et al. vs. Stockholm MRI grading system | 0.292 |
| MRI grading system of Firsching et al. vs. MRI grading system of Abu Hamdeh et al. | 0.483 |
| MRI grading system of Firsching et al. vs. Stockholm MRI grading system | 0.153 |
| MRI grading system of Abu Hamdeh et al. vs. Stockholm MRI grading system | 0.336 |

Supplemental Table 7. Differences in discrimination between different grading systems of traumatic axonal injuries

This table presents the results obtained using De long’s test when examining whether there were any statistically significant differences in the AUC, a measure of discrimination, between the different TAI grading systems. The univariate logistic regression models used to assess discrimination were fit using the dichotomised Glasgow outcome scale was used as the dependent variable. Abbreviations: Core = Core variables, CT = Computed tomography, MRI = Magnetic resonance imaging.
