# Supplementary material for: Extended Analysis of Axonal Injuries Detected Using Magnetic Resonance Imaging in Critically Ill Traumatic Brain Injury Patients
Source: J Neurotrauma. 2022 Jan 11;39(1-2):58–66. doi: 10.1089/neu.2021.0159 (PMC8785713; doi:10.1089/neu.2021.0159)
Supplement: Supplemental data [file Supp_FigS7.docx]

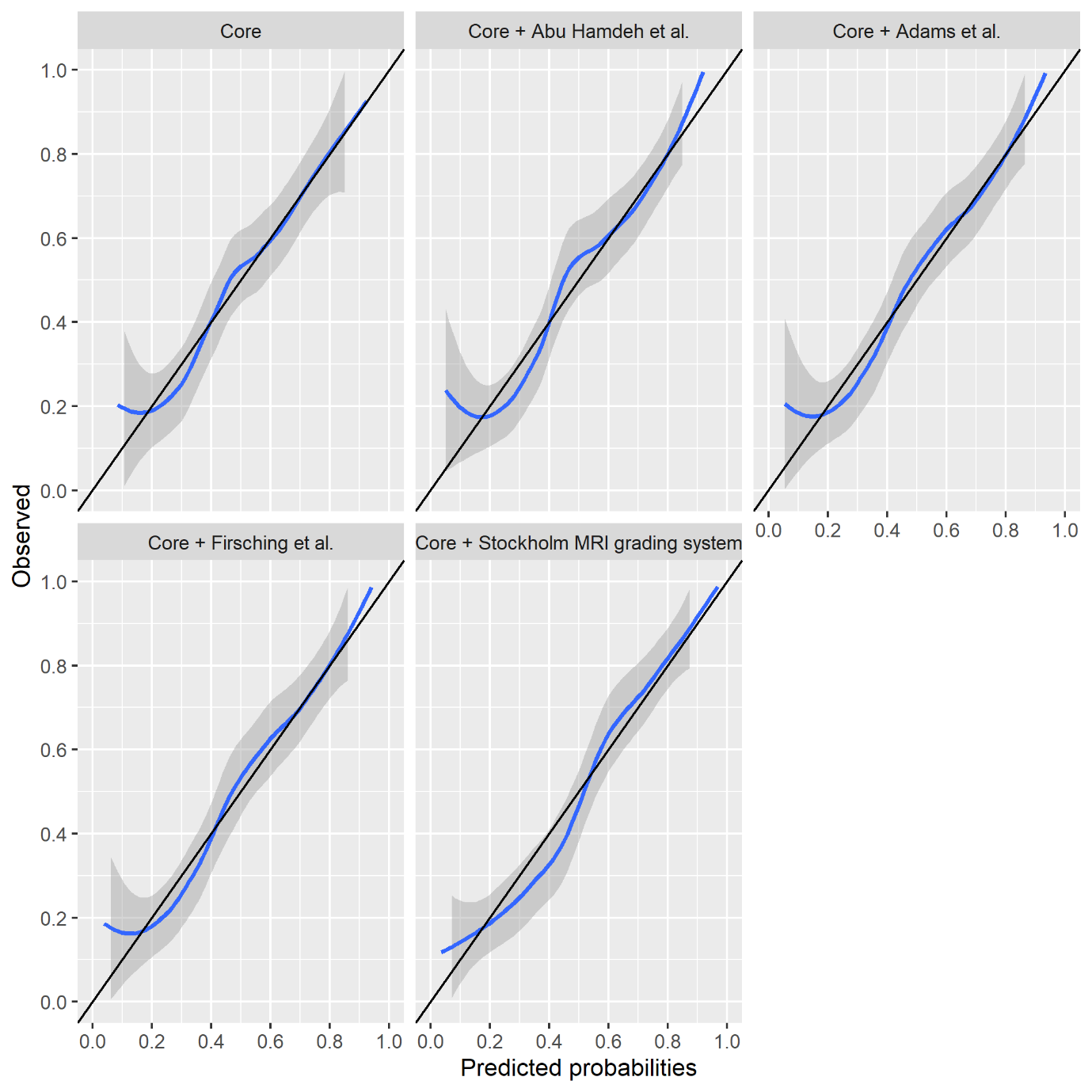


**Supplemental figure 7.** Calibration plots, computed from the results of multivariate logistic regression models comparing the magnetic resonance imaging (MRI) -based traumatic axonal injury (TAI) grading systems of Adams *et al*., Firsching *et al*., Abu Hamdeh *et al*. and the Stockholm MRI grading system with the core variables, consisting of the Glasgow coma scale at admission, pupillary reactivity and age. The dichotomised Glasgow outcome scale was used as the dependent variable.
