# Supplementary material for: Extended Analysis of Axonal Injuries Detected Using Magnetic Resonance Imaging in Critically Ill Traumatic Brain Injury Patients
Source: J Neurotrauma. 2022 Jan 11;39(1-2):58–66. doi: 10.1089/neu.2021.0159 (PMC8785713; doi:10.1089/neu.2021.0159)
Supplement: Supplemental data [file Supp_TableS2.docx]

| scanner | Time period | TR (ms) | TE (ms) | Flip angle |
| --- | --- | --- | --- | --- |
| GE Signa (1.5T) | 2005-2006 | 8500 | 112 | 90° |
|  | 2005-2007 | 8500 | 111 | 90° |
|  | 2006-2008 | 8500 | 116 | 90° |
|  | 2007-2009 | 8500 | 123 | 90° |
|  | 2008-2010 | 8500 | 132 | 90° |
| Siemens Avanto (1.5T) | 2010-2011 | 3100 | 78 | 90° |
|  | 2010-2015 | 3800 | 78 | 90° |
|  | 2015-2018 | 5900 | 74 | 180° |
| GE signa (3T) | 2018-2019 | 6670 | 83 | 90° |

**Supplemental Table 2.** **MRI protocol for the DWI sequence.**

A table summarising the parameters used for Diffusion weighted imaging. b-values of 0 and 1000 sec/mm^2^ were used for all DWI protocols. Abbreviations: DWI = Diffusion weighted imaging, MRI = Magnetic Resonance imaging, GE = General Electric, TR = Repetition time, TE = Echo time.
