# Supplementary material for: Extended Analysis of Axonal Injuries Detected Using Magnetic Resonance Imaging in Critically Ill Traumatic Brain Injury Patients
Source: J Neurotrauma. 2022 Jan 11;39(1-2):58–66. doi: 10.1089/neu.2021.0159 (PMC8785713; doi:10.1089/neu.2021.0159)
Supplement: Supplemental data [file Supp_FigS2.docx]

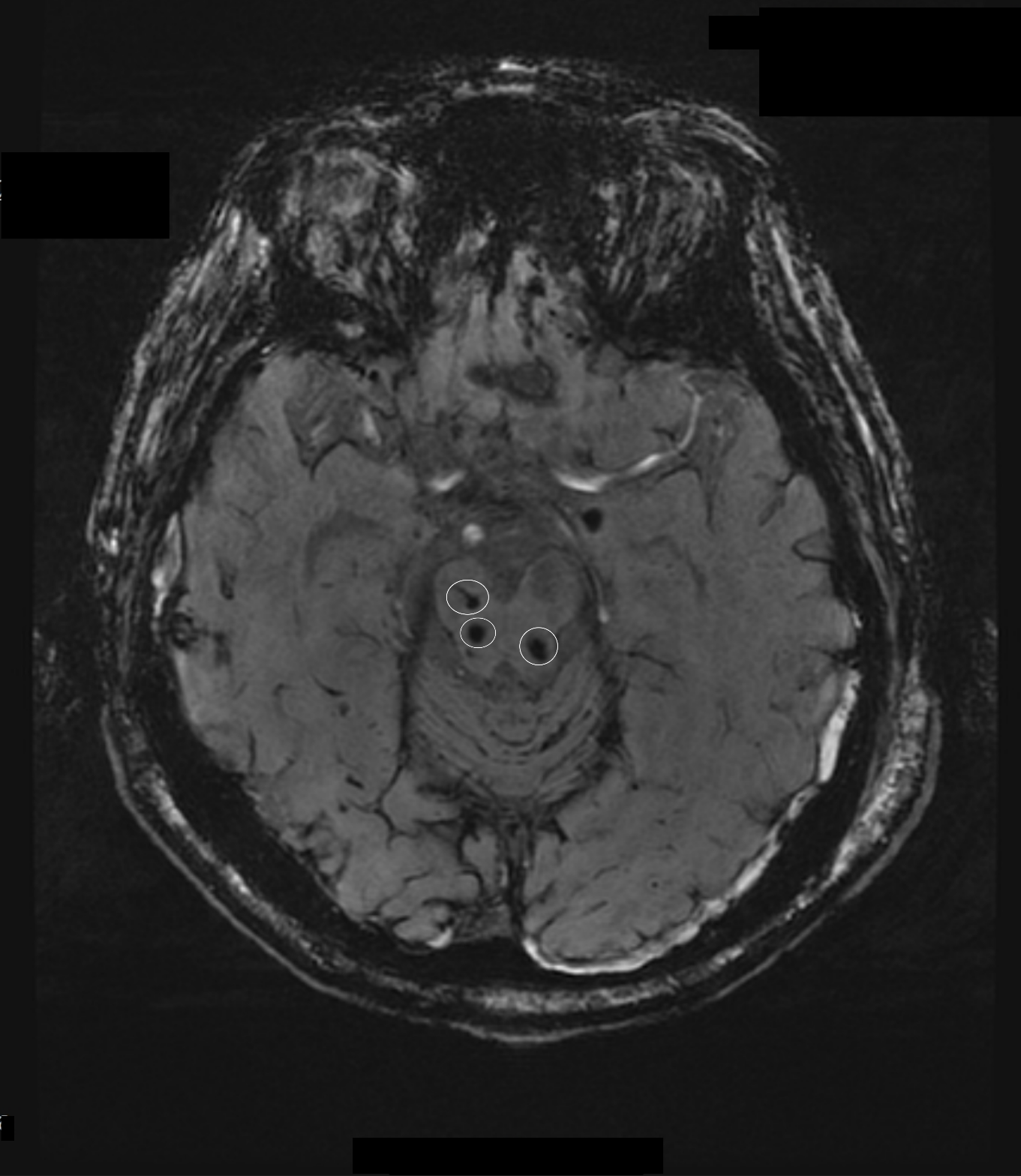


Supplemental Figure 2. Susceptibility-sensitive sequences.

Traumatic axonal injuries detected using susceptibility-weighted imaging (SWI) in the midbrain, marked with white circles.
