# Supplementary material for: Extended Analysis of Axonal Injuries Detected Using Magnetic Resonance Imaging in Critically Ill Traumatic Brain Injury Patients
Source: J Neurotrauma. 2022 Jan 11;39(1-2):58–66. doi: 10.1089/neu.2021.0159 (PMC8785713; doi:10.1089/neu.2021.0159)
Supplement: Supplemental data [file Supp_DataS1.docx]

**Supplemental information. Additional details regarding feature selection and machine learning**

*Feature selection and Machine learning*

Before the machine learning analysis, the data underwent several pre-processing steps. The Glasgow Coma Scale (GCS), although theoretically an ordinal categorical variable, was treated as a numeric variable for the purpose of this analysis, in accordance with previous publications in this field^1^. We normalised the numeric variables age and GCS, such that each variable had a standard deviation of 1 and a mean equal to 0. The categorical variables included all traumatic axonal injuries (TAI) described in Supplemental Table 4, which were separated by MRI pulse sequence, in addition to the pupillary response on admission. The aforementioned categorical variables underwent one-hot encoding, which entails converting each categorical variable into “N-1” binary variables, where “N” denotes the number of categories present in the original categorical variable. Next, we split the data into training- and test datasets. The training dataset consisted of 2/3 of the data, whereas the test dataset consisted of the remaining data. We used the training dataset during feature selection, training and hyperparameter tuning, whereas the test dataset was only used in the subsequent validation of those results.

In order to select TAI which may aid in predicting long-term functional outcome following TBI, we used the metaheuristic genetic algorithm (GA) on the training dataset.^2^ A GA is a search algorithm and an optimisation method that is based on the concept of natural selection, and which is commonly used to solve complex search and optimisation problems. We used the R-package “GA” to run the GA.^3^ We maintained the population size at 50 for each successive generation, while the crossover rate and the mutation rate were set to 0.8 and 0.1, respectively. The initial population consisted of approximately 30% of all available variables, which were sampled randomly. The maximum number of iterations was set to 2,000 for the GA. The GA was programmed to terminate if there was no improvement in fitness in the span of 200 iterations. We evaluated the predictive performance of each consecutive generation of the GA using a machine learning algorithm called random forest, with the dichotomised GOS as the dependent variable. The number of variables, which were randomly sampled as candidates for each split of the random forest, was maintained at the square root of the total number of variables. We selected the number of trees grown with each run of the random forest using repeated 10-fold cross-validation, with 5 repetitions (cross-validation is discussed in greater detail below). The metric used to assess population fitness was a so-called “desirability function”.^4^ Desirability functions enable the simultaneous optimisation of multiple response measures. In the current study, the desirability function consisted of the area under the receiver operating characteristic curve (AUC), as well as the inverse of the number of variables used in fitting the model. The AUC, which the random forest was used to estimate for each chromosome during each successive iteration of the GA, is a statistical estimate of discrimination. Thus, the desirability function penalises overly complicated solutions, while simultaneously ensuring that the GA maximises the discrimination between favourable and unfavourable outcomes.

Despite being a highly robust method, the GA is nevertheless susceptible to overfitting^5^. Therefore, to validate that the solution proposed by the GA is generalisable, a random forest model was validated on the test dataset. Random forest is a machine learning algorithm. We used the R package “randomForest” to run the random forest models. Training and hyperparameter tuning of the random forest were carried out using repeated 10-fold cross-validation with 10 repetitions using the training dataset. Hyperparameter tuning was done using a grid search protocol. The metric for model performance, which we used in conjunction with the cross-validation to estimate the best fitting model, was the AUC.

The performance of the model in predicting long-term functional outcomes in unseen data was ultimately evaluated on the test dataset, which was not used in feature selection or the training of the random forest model during the previous steps.^6,7^ The random forest model’s ability to discriminate between favourable and unfavourable outcomes in the test dataset was evaluated using the AUC. We also plotted receiver operating characteristic (ROC) curves for each model, to provide a visual representation of the differences in model performance.

References:

1. MRC CRASH Trial Collaborators, Perel, P., Arango, M., Clayton, T., Edwards, P., Komolafe, E., Poccock, S., Roberts, I., Shakur, H., Steyerberg, E., and Yutthakasemsunt, S. (2008). Predicting outcome after traumatic brain injury: practical prognostic models based on large cohort of international patients. BMJ 336, 425–429.

2. Holland, J.H. (John H. (1992). Adaptation in natural and artificial systems : an introductory analysis with applications to biology, control, and artificial intelligence. MIT Press, 211 p.

3. Scrucca, L. (2013). GA: A package for genetic algorithms in R. J. Stat. Softw. 53, 1–37.

4. Derringer, G., and Suich, R. (1980). Simultaneous Optimization of Several Response Variables. J. Qual. Technol. 12, 214–219.

5. Santos, E.M. Dos, Sabourin, R., and Maupin, P. (2008). Overfitting cautious selection of classifier ensembles with genetic algorithms.

6. Burges, C.J.C. (1998). A Tutorial on Support Vector Machines for Pattern Recognition. Data Min. Knowl. Discov. 2, 121–167.

7. Boser, B.E., Guyon, I.M., and Vapnik, V.N. (1992). A training algorithm for optimal margin classifiers., in: *Proceedings of the Fifth Annual Workshop on Computational Learning Theory - COLT ’92*. New York, New York, USA: ACM Press, pps. 144–152.
